# Improving genome-wide mapping of nucleosomes in *Trypanosome cruzi*.

**DOI:** 10.1371/journal.pone.0293809

**Published:** 2023-11-21

**Authors:** Paula Beati, Milena Massimino Stepñicka, Salomé C. Vilchez Larrea, Pablo Smircich, Guillermo D. Alonso, Josefina Ocampo

**Affiliations:** 1 Instituto de Investigaciones en Ingeniería Genética y Biología Molecular, Consejo Nacional de Investigaciones Científicas y Técnicas, Buenos Aires, Argentina; 2 Departamento de Fisiología, Biología Molecular y Celular, Facultad de Ciencias Exactas y Naturales, Universidad de Buenos Aires, Buenos Aires, Argentina; 3 Laboratorio de Bioinformática, Departamento de Genómica, Instituto de Investigaciones Biológicas Clemente Estable (IIBCE), Montevideo, Uruguay; 4 Sección Genómica Funcional, Facultad de Ciencias, Universidad de la República (UdelaR), Montevideo, Uruguay; Universidade Federal de Minas Gerais, BRAZIL

## Abstract

In *Trypanosoma cruzi* DNA is packaged into chromatin by octamers of histone proteins that form nucleosomes. Transcription of protein coding genes in trypanosomes is constitutive producing polycistronic units and gene expression is primarily regulated post-transcriptionally. However, chromatin organization influences DNA dependent processes. Hence, determining nucleosome position is of uppermost importance to understand the peculiarities found in trypanosomes. To map nucleosomes genome-wide in several organisms, digestion of chromatin with micrococcal nuclease followed by deep sequencing has been applied. Nonetheless, the special requirements for cell manipulation and the uniqueness of the chromatin organization in trypanosomes entails a customized analytical approach. In this work, we adjusted this broadly used method to the hybrid reference strain, CL Brener. Particularly, we implemented an exhaustive and thorough computational workflow to overcome the difficulties imposed by this complex genome. We tested the performance of two aligners, Bowtie2 and HISAT2, and discuss their advantages and caveats. Specifically, we highlight the relevance of using the whole genome as a reference instead of the commonly used Esmeraldo-like haplotype to avoid spurious alignments. Additionally, we show that using the whole genome refines the average nucleosome representation, but also the quality of mapping for every region represented. Moreover, we show that the average nucleosome organization around trans-splicing acceptor site described before, is not just an average since the same chromatin pattern is detected for most of the represented regions. In addition, we extended the study to a non-hybrid strain applying the experimental and analytical approach to Sylvio-X10 strain. Furthermore, we provide a source code for the construction of 2D plots and heatmaps which are easy to adapt to any *T*. *cruzi* strain.

## Introduction

In eukaryotes the nuclear DNA is packaged into chromatin, which is organized in repetitive units called nucleosomes. These basic units are composed of two of each of the canonical histones: H2A, H2B, H3, and H4, constituting a protein core around which a stretch of ∼147 bp of DNA is wrapped [1].

*In vivo*, nucleosomes are regularly spaced by linker DNA. There seems to exist a correlation between DNA accessibility and the linker length, suggesting that nucleosome spacing is the primary determinant of gene regulation [2]. Additionally, a primary role of nucleosomes in controlling transcription was recently proposed [3]. Hence, studying nucleosome positioning is of paramount relevance to understand how genomic DNA is packed and how DNA-dependent processes are regulated.

In *T*. *cruzi*, chromatin is also organized in repetitive units [4], but shows some unusual characteristics compared to other organisms. Among these peculiarities, 30 nm fibers do not form *in vitro* [5] and chromatin does not condense into chromosomes during mitosis [6]. This phenomenon is suspected to be due to the unique characteristic of its histone H1, which lacks the globular domains typically present in other eukaryotes [7]. Besides, the infective and non-infective parasite life forms display different levels of chromatin condensation during interphase [5], differential susceptibility to DNaseI [8], and some differences in the nucleosome landscape [9]. Additionally, the histones of trypanosomes are the least conserved among all eukaryotic histones studied so far, differing from other organisms in size, sequence, and charge [7, 10]. Given all these distinctive properties and the potential implications for key cellular processes, a dedicated study of *T*. *cruzi* chromatin organization is required.

Several methods for genome-wide high-resolution chromatin profiling have been developed but not all of them are easy to adapt to every organism under study [11, 12]. Despite having some limitations and caveats, the most widely used method, applied first in yeast, is the digestion of chromatin with micrococcal nuclease (MNase) followed by deep sequencing using paired-end technology (MNase-seq) [13]. In the last few years, a huge number of nucleosome maps of numerous organisms including *T*. *brucei* and *Leishmania major* were generated [14–16]. More recently, a good experimental setup for epimastigotes and trypomastigotes of the *T*. *cruzi* CL Brener strain was published [9]. However, given the hybrid nature of the CL Brener strain several analytical challenges remained unsolved.

Despite the large number of informatic tools currently available [17], some careful considerations when applying the informatics workflow are required. All *T*. *cruzi* strains are diploid but there are two kinds: (1) clonal strains, harboring equivalent homologous chromosomes; and (2) hybrid strains, carrying one copy of each chromosome from two substantially different parental strains. The latter adds some complexity to the data analysis. In this sense, numerous genomic studies are performed using the reference hybrid strain CL Brener, composed of the Esmeraldo-like and non-Esmeraldo-like haplotypes. However, for data processing the community working in the field sometimes uses just one haplotype as reference genome for simplicity. In this work, we present a detailed protocol for nucleosome preparation and genome-wide mapping for *T*. *cruzi* epimastigotes. Moreover, we have performed a thorough analysis to find the most suitable steps to cope with the special challenges imposed by the hybrid nature of CL Brener, emphasizing the importance of using its whole genome as a reference. As proof of concept, we show that only when using the whole genome as a reference, we avoid artefactual signals, and improved the detection of nucleosome occupancy. Moreover, we show that the average nucleosome organization around trans-splicing acceptor site described before, is not just an average since the same chromatin pattern is detected for most of the represented regions. Additionally, we applied the experimental and analytical approach to Sylvio-X10 strain showing that the method can be extended to a non-hybrid strain. Furthermore, we provide an itemized pipeline for analytical processing and the scripts for the construction of 2D plots and heatmaps which are easy to adapt to any *T*. *cruzi* strain.

## Materials and methods

“The protocol described in this peer-reviewed article is published on protocols.io, DOI: https://doi.org/10.17504/protocols.io.6qpvr4w83gmk/v2 and is included for printing as S1 File with this article.

## Results

### General outline

**Fig 1:**
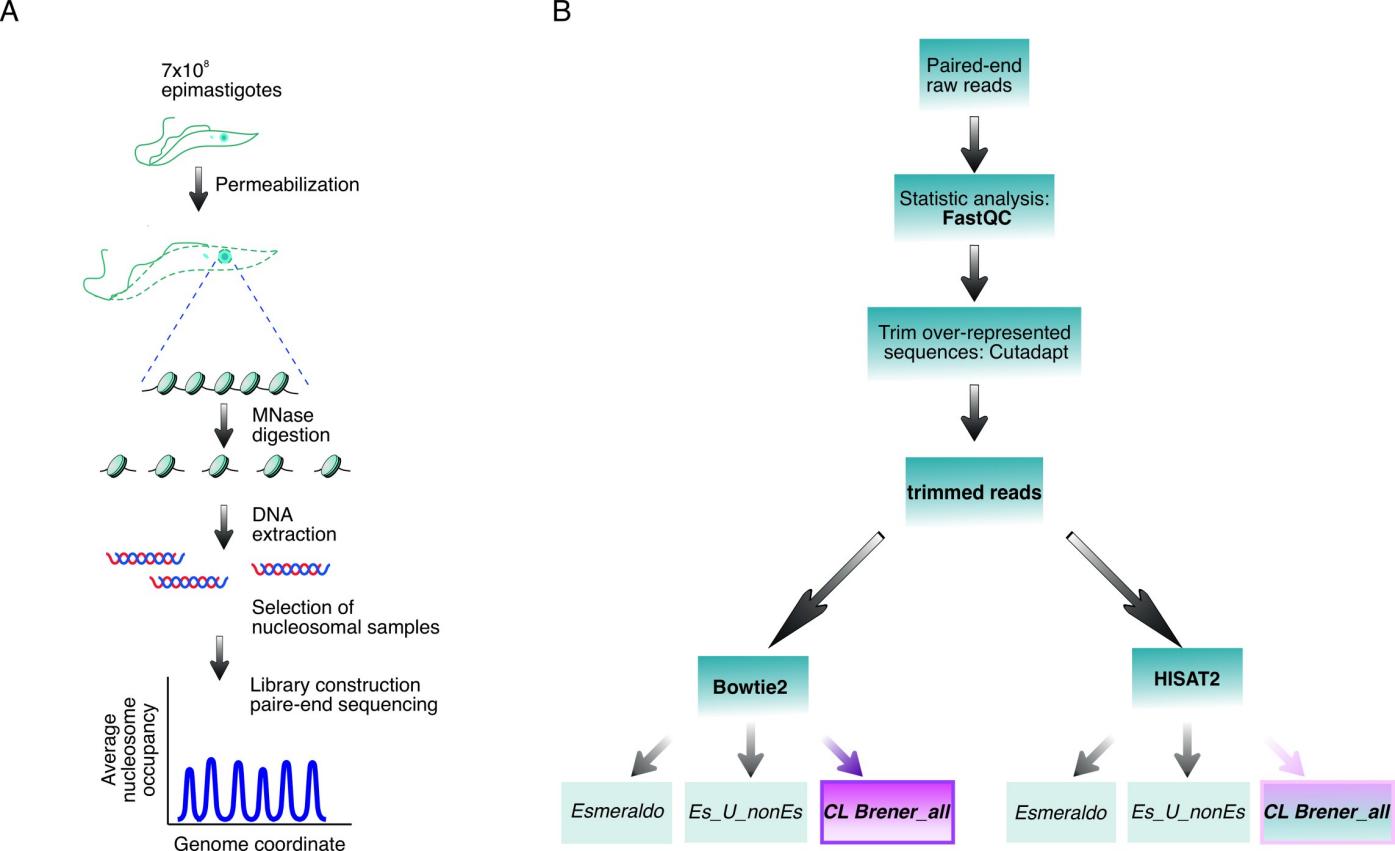
Experimental and informatics workflow. **(A)** Schematic representation of the experimental workflow. **(B)** Schematic workflow of the pipeline used to analyze the MNase-seq data. The use of the *CL Brener_all* as reference genome is suggested independently of the aligner of choice and is the most critical analytical choice.

The main objective of this method is to obtain fragments of ∼147 bp of DNA, which are protected from MNase digestion by the presence of the nucleosome core. Exponentially growing *T*. *cruzi* epimastigotes are collected, permeabilized and chromatin is digested with MNase. After stopping the reaction, DNA is extracted, and the level of digestion is checked in an agarose gel. MNase-seq libraries are constructed as described [18, 19]. Due to the peculiarities of *T*. *cruzi* and the unprecedented analysis with this parasite, we followed the standard bioinformatic analysis described for yeast [20, 21]. Nevertheless, we tested alternative strategies at every step of the way to find the most suitable path (details below). Experimental and informatics workflows are shown in Fig 1A and 1B respectively.

### Selecting the best mononucleosomal sample to proceed with

We grew the parasites and performed MNase digestion as described before [22] with modifications (see S1 File for details). To determine the extent of digestion 10 μl of each MNase-digested sample was analyzed in a 2% agarose gel next to the PCR DNA Marker (New England Biolabs, Ipswich, MA, US). An ideal sample should have more than 80% of the DNA represented in the mononucleosome band and a faint band in the di-nucleosome band as shown in S1 Fig in S3 File. In poorly digested samples a ladder of bands is observed, and only a small fraction of the total DNA is contained in the mononucleosome fraction. This mononucleosome band is longer than 150 bp due to poor trimming. Moreover, early digestion is biased to AT-rich DNA sequences since they are preferentially cut by MNase and the protected regions may include non-histone DNA binding complexes [20]. On the other hand, in over digested samples, faint bands appear underneath the mononucleosome band due to excessive trimming of the core particle. It is still possible to tell the position of the remaining nucleosomes, but some of them will be lost. Therefore, nucleosome occupancy maps will not be accurate. Hence, a compromise is required, keeping in mind that ∼80% of the DNA should be contained in the mononucleosome band. Testing an aliquot of the selected sample in an Agilent Bioanalyzer 2100 expert with DNA 1000 (Agilent Scientific Instruments, Santa Clara, CA, US), which provides electropherograms and gel-like images, might help to make the correct decision, but is not indispensable. In any case, the precise length distribution of the chosen sample will be verified after paired-end sequencing as described below.

### Nucleosome preparation and deep sequencing

We established a straightforward protocol for nucleosome preparation from epimastigotes of any *T*. *cruzi* strain as described in S1 File. We achieved good quality nucleosome preparation for replicated experiments of CL Brener strains. The agarose gel for a replicated experiments and the number of total paired-reads counted after aligning the replicated data to the reference genome, as well as the reproducibility of the replicated experiments are shown in S2 Fig in S3 File.

### Bioinformatic analysis

Basic bioinformatic analysis is straightforward when analyzing data from model organisms such as yeast, fly or mouse. However, *T*. *cruzi* presents so many peculiarities that extensive work is required. To find the most appropriate steps for *T*. *cruzi* analysis, several points of the standard workflow were tested. First, we analyzed whether it was necessary or not to trim the overrepresented sequences from the fastq files. Second, the capacity to achieve the most accurate alignment was compared between the widely used aligner Bowtie2 [23], and the newer tool HISAT2 [24]. Third, alternative reference genomes were tested, including the commonly used Esmeraldo-like haplotype, a combination of both haplotypes (Esmeraldo-like + non-Esmeraldo-like haplotypes) and a more comprehensive one including the non-designated regions (Esmeraldo-like + non-Esmeraldo-like haplotypes + "extra") (Fig 1B). Once the most reliable workflow was established, we generated the pipeline to obtain length distribution histograms of DNA sequences, bigwig files, average plots, and particularly 2D plots and heatmaps specially customized for this organism (see details below). Additionally, a source code easy to adapt to any *T*. *cruzi* strain is available at (https://github.com/paulati/nucleosome).

### Length distribution histograms

**Fig 2:**
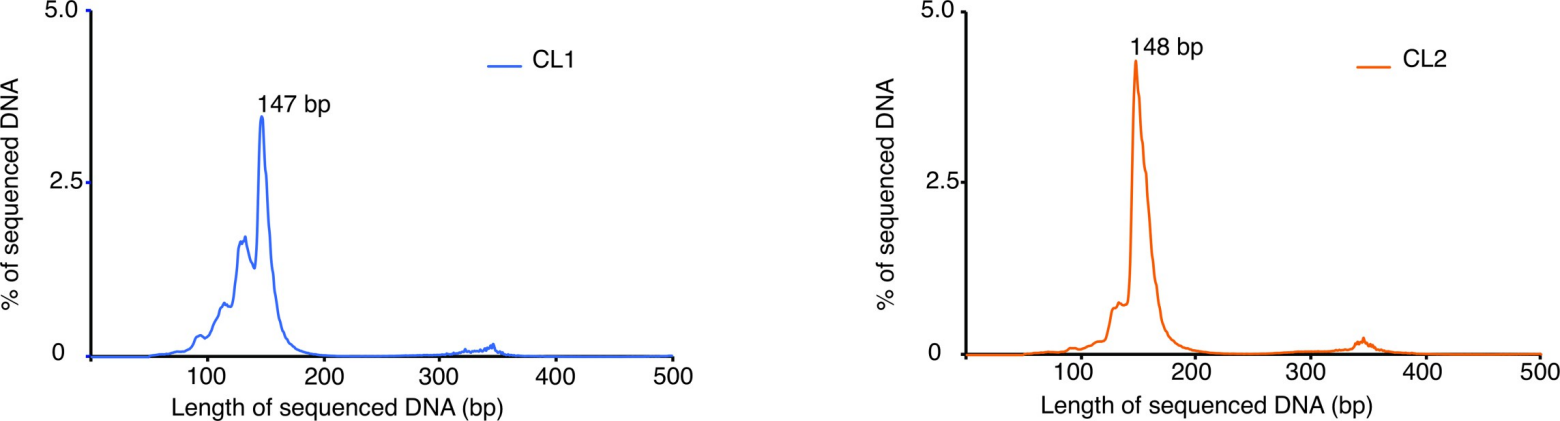
Length distribution of sequenced DNA. Length histogram for all nucleosomal DNA sequenced for replicate 1 (left panel) and replicate 2 (right panel) respectively.

Once we aligned the paired-reads to the reference genome, we checked the level of digestion achieved for the sequenced samples by plotting a length distribution histogram, as previously described [13]. Both samples from the two replicated experiments present a peak around the nucleosome size ∼147–148 bp showing that we obtained the right level of digestion (Fig 2). It is worth noting that to compare different samples, a good criterion is to use samples with similar length distribution histograms, indicating similar levels of digestion.

### Selection of the alignment tool and choice of the best reference genome for the CL Brener strain

To find the most suitable pipeline to analyze CL Brener data, we tested the alignment of raw and trimmed reads either to *Esmeraldo*, *Es_U_nonEs* or *CL Brener_all* genomes using Bowtie2 and HISAT2. Bowtie2 is the most widely used aligner and is considered a multi-purpose tool, while HISAT2, was originally designed for RNA-seq analysis. HISAT2 bears a few optimizations including improving the accuracy of short-read alignment. Therefore, we pursued in parallel both strategies (Fig 1B). We compared the output statistics of the alternative alignments for both replicated experiments, which are summarized in Table 1 and S1 Table in S3 File respectively.

**Table 1:** Statistics of alignments.

*CL Brener*	Bowtie2	trimmed reads	genome	times	time	> time	overall
			*Esmeraldo*	46.63	22.93	30.44	53.37
			*Es_U_nonEs*	36.42	10.66	53.1	63.76
			*CL Brener_all*	28.89	9.05	62.06	71.11
		raw reads	genome	0 times	time	> time	overall
			*Esmeraldo*	47.19	22.68	30.13	52.81
			*Es_U_nonEs*	36.67	10.36	52.96	63.33
			*CL Brener_all*	29.58	8.9	61.52	70.42
	HISAT2	trimmed reads	genome	0 times	1 time	> time	overall
			*Esmeraldo*	47.01	41.65	11.34	52.99
			*Es_U_nonEs*	38.07	45.65	16.28	61.93
			*CL Brener_all*	36.42	40.79	22.8	63.58
		raw reads	genome	0 times	1 time	> time	overall
			*Esmeraldo*	47.58	41.2	11.21	52.42
			*Es_U_nonEs*	38.74	45.16	16.1	61.26
			*CL Brener_all*	37.1	40.35	22.55	62.9

The percentage of alignments for the different paths tested with replicate 1 is summarized. Values obtained when using trimmed or raw reads aligned to the alternative genomes are shown.

The first challenge was to test whether trimming the overrepresented sequences was necessary, and we observed that in general it had a minimum effect for both aligners. When using Bowtie2, trimming raw reads only led to a tiny improvement in the overall alignment in less than 1% with any genome used (from 70.42% to 71.11% when using *CL Brener_all* genome; from 63.33% to 63.76% when using *Es_U_nonEs* genome; or from 52.81% to 53.37% when using *Esmeraldo* genome), by slightly increasing the number of reads that align to the genome only one time, or more than once, accompanied by a small reduction in the number of reads that align zero times, consistent with the fact that most of the trimmed sequences correspond to adaptors or primer sequences. A similar trend is observed when using HISAT2 (Table 1 and S1 Table in S3 File).

The second step was to evaluate the most suitable genome to use as a reference. Due to the hybrid nature of CL Brener, its two haplotypes are annotated separately: Esmeraldo-like and non-Esmeraldo-like, each of them having 41 chromosomes, called S and P chromosomes respectively. An additional complication is that part of the genome is not assigned either to the Esmeraldo-like or to the non-Esmeraldo-like haplotypes and is annotated separately, “extra”. In a previous high throughput study using CL Brener, the output data sets were aligned only to the genome of the Esmeraldo-like haplotype for simplicity [9]. To test the noise introduced by this simplification, we tested two additional reference genomes: *Es_U_nonEs* and *CL Brener_all* for comparison. For Bowtie2, the use of the *CL Brener_all* genome as a reference improved the overall alignment by ∼7% with respect to the *Es_U_nonEs* genome (from 63.76% to 71.11%) and by ∼18% with respect to the *Esmeraldo* genome (from 53.37% to 71.11%) when using trimmed reads at the expense of an increment in the number of reads that align more than once. This observation is associated with a significant reduction in the number of reads that align zero times, suggesting that the fraction of reads aligned only when using *CL Brener_all* belongs to regions of the genome that are not assigned to any of the haplotypes, probably due to their repetitive nature. While for HISAT2, using the *CL Brener_all* as the reference genome improves by only ∼1.65% the overall alignment with respect to using *Es_U_nonEs* (from 61.93% to 63.58%), the improvement relative to using the *Esmeraldo* genome was ∼10% (from 52.99% to 63.58%) based on reads that align more than once.

Third, comparing Bowtie2 with HISAT2, the overall alignment is better when using Bowtie2 by ∼7.5% when using the *CL Brener_all* genome (from 63.58% to 71.11%), by ∼2% when using the *Es_U_nonEs* genome (from 61.49% to 63.76%), but the difference is insignificant when using the *Esmeraldo* genome (from 52.99% to 53.37%). Although the genome choice seems to be unimportant for HISAT2, when comparing *CL Brener_all* and *Es_U_nonEs*, using the *CL Brener_all* genome avoids artefactual alignments (discussed below). The biggest improvement of using HISAT2 over Bowtie2 is the increment in the number of reads that align only once by ∼32% (from 9.05% to 40.79%) when aligning to *CL Brener_all* genome, by ∼35% when aligning to *Es_U_nonEs* genome (from 10.66% to 45.65%), and by ˜19% when using *Esmeraldo* (from 22.93% to 41.65%), consistent with HISAT2 being more efficient to detect uniquely aligned reads with any reference genome tested.

To summarize, trimming has a minimal effect in any path followed. However, considering that the removed reads are spurious, it is a good advice to cut them out to avoid any artefactual noise in later analysis. Overall, using Bowtie2 with *CL Brener_all* genome is a good choice and allows a better overall alignment than HISAT2. However, HISAT2 outstrips Bowtie2 by being more efficient to solve the alignment of reads at multiple places in the genome, particularly relevant when working with a hybrid strain (Table 1 and S1 Table in S3 File). Finally, using *CL Brener_all* as a reference genome instead of *Es_U_nonEs*, or just *Esmeraldo* constitutes the critical point of the workflow (discussed in detail below).

### Using *CL Brener_all* genome as a reference prevents spurious alignments

To make a visual inspection of how nucleosomes are organized in a particular region or loci of interest, we generated bigwig files containing either nucleosome occupancy maps or nucleosome position maps and inspected them in the Integrative Genome Viewer (IGV). This visualization facilitates the comparison between samples and the examination of nucleosome position or nucleosome occupancy maps against the chromosome coordinates. In S3 Fig in S3 File, we show nucleosome occupancy and nucleosome dyads for one representative region of CL Brener replicated experiments.

**Fig 3:**
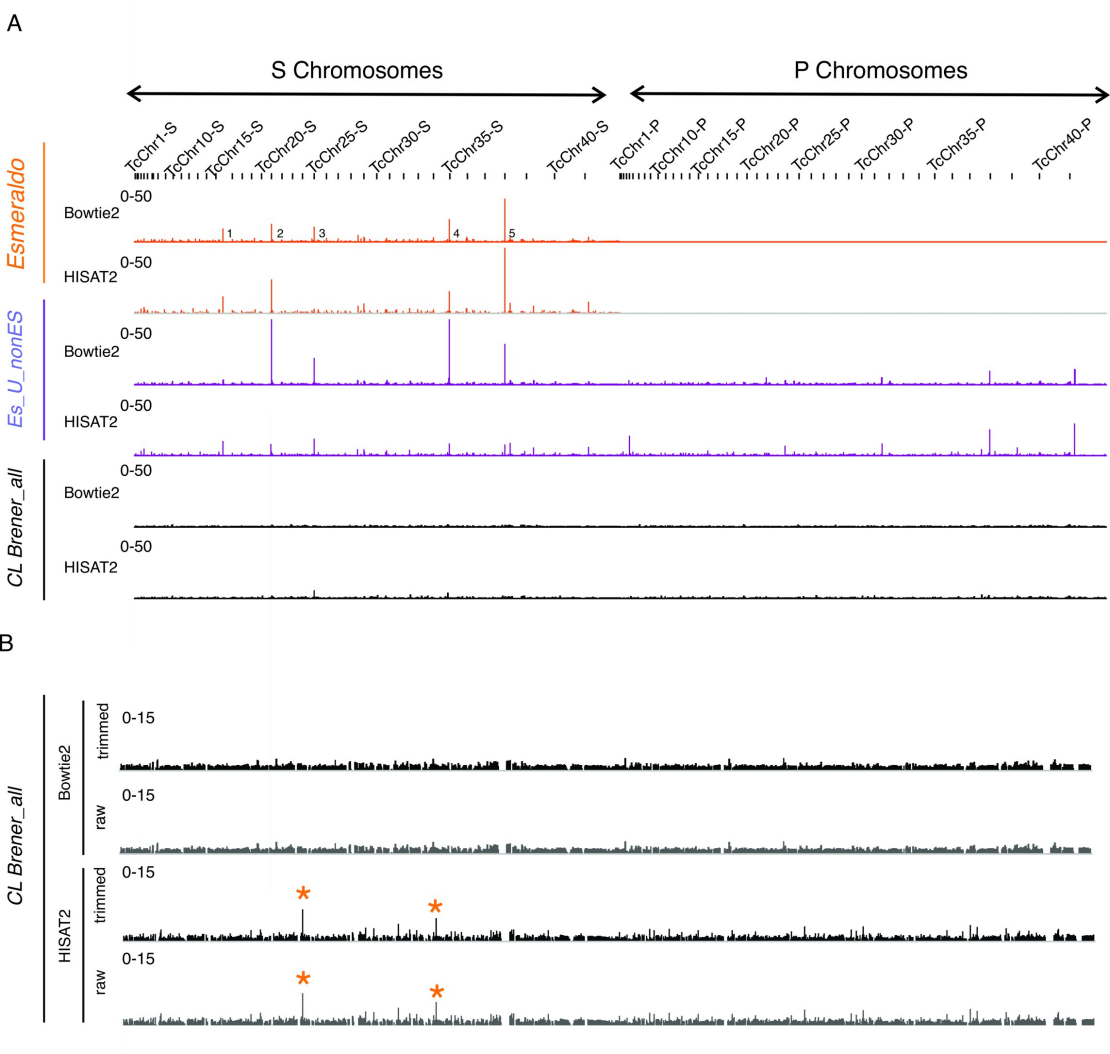
Using the *CL Brener_all* genome prevents spurious alignments. **(A)** IGV image for normalized nucleosome occupancy maps for the whole genome generated with trimmed reads from one representative data set (replicate 1) aligned to the *Esmeraldo* (orange), *Es_U_nonEs* (purple) and *CL Brener_all* (black) genomes respectively either with Bowtie2 or HISAT2. Numbers 1–5 indicate the positions of the unusually high regions, being their genomic locations as follow: 1: TcChr15-S:394,562–407,352; 2: TcChr20-S:533,008-533-199; TcChr20-S:609,542–611,666 and TcChr20-S:655,814–656,697; 3:TcChr24-S:776,462–779,922, 4:TcChr34-S:1,062,855–1,065,609 and 5: TcChr37-S:1,198,569 and TcChr37-S:1,354,149–1,355,690". **(B)** Magnified IGV image for the nucleosome occupancy maps generated from trimmed and raw reads aligned to the *CL Brener_all* genome. Orange (*) indicates the main artefacts introduced when using HISAT2, coincidental with the signals numbered 3 and 4 in panel A respectively.

Additionally, we performed a comparison of the nucleosome occupancy generated from the different alignments described above using IGV. When we used either *Esmeraldo* or *Es_U_nonEs* as reference genomes with any aligner tested, we observed a few regions of extremely high nucleosome density (Figs 3A, S4A, S5A and S5B in S3 File). These unusually high signals, that in general correspond to the most distal regions of the chromosomes, were smoothed out almost completely when using *CL Brener_all* as reference genome. Both aligners seem to work very well with the *CL Brener_all* genome; but when using HISAT2, we observed a couple of regions with higher nucleosome density (Figs 3B, S4B and S5C in S3 File). These unusually high-density regions are numbered from 1–5 and we provide the genomic locations of the more prominent signals within them in the figure legend. One of these unusually high signals was detected at the Mucin-associated surface protein (MASP) locus, a multi copy gene family. Finally, trimming had a minimum effect as observed in Fig 3B and S5C Fig in S3 File.

### Prediction of trans-splicing acceptor sites (TAS)

For genomic analysis, manual viewer inspection of the data sets is not enough, it should be complemented with average analysis of the whole genome which is normally plotted relative to a reference point. For model organisms such as yeast, the average nucleosome occupancy or the average dyad occupancy for every gene in the genome is usually represented relative to the transcription start sites **(TSSs)** or +1 nucleosome. This representation results in a prototypical chromatin organization with peaks at nucleosome midpoints and depressions at linker DNA. The peaks are regularly spaced and phased relative to the TSS and preceded by a nucleosome depleted region upstream of the +1 nucleosome [25–27]. This chromatin organization is broadly conserved among different organisms [28–30]. However, the scenario is completely different for trypanosomes.

In trypanosomatides, this genomic analysis is extremely challenging. On one hand, most of the genes are transcribed into polycistronic transcription units **(PTUs)** with no clear TSS. Each PTU matures into monocistronic mRNA by the addition of a 39 bp sequence denominated splice leader at the 5’ untranslated region **(5’UTR)** and a 3’ untranslated region **(3’UTR)** polyadenylation in a process known as trans-splicing [31].

In *L*. *major*, *T*. *brucei* and recently in *T*. *cruzi* poor chromatin organization was observed consistent with the fact that the genome is being constantly transcribed. In these organisms, nucleosomes are organized around the trans-splicing acceptor site **(TAS)** [9, 14–16]. In this work, we predicted the TAS as described in methods (S1 File) by using the UTRme predictor [32]. We performed the TAS prediction only for the Esmeraldo-like haplotype and we obtained the genomic coordinates for those with best score (S2 File).

### Proof of concept: Nucleosome signals around TAS are significantly improved when using *Cl Brener_all* genome

**Fig 4:**
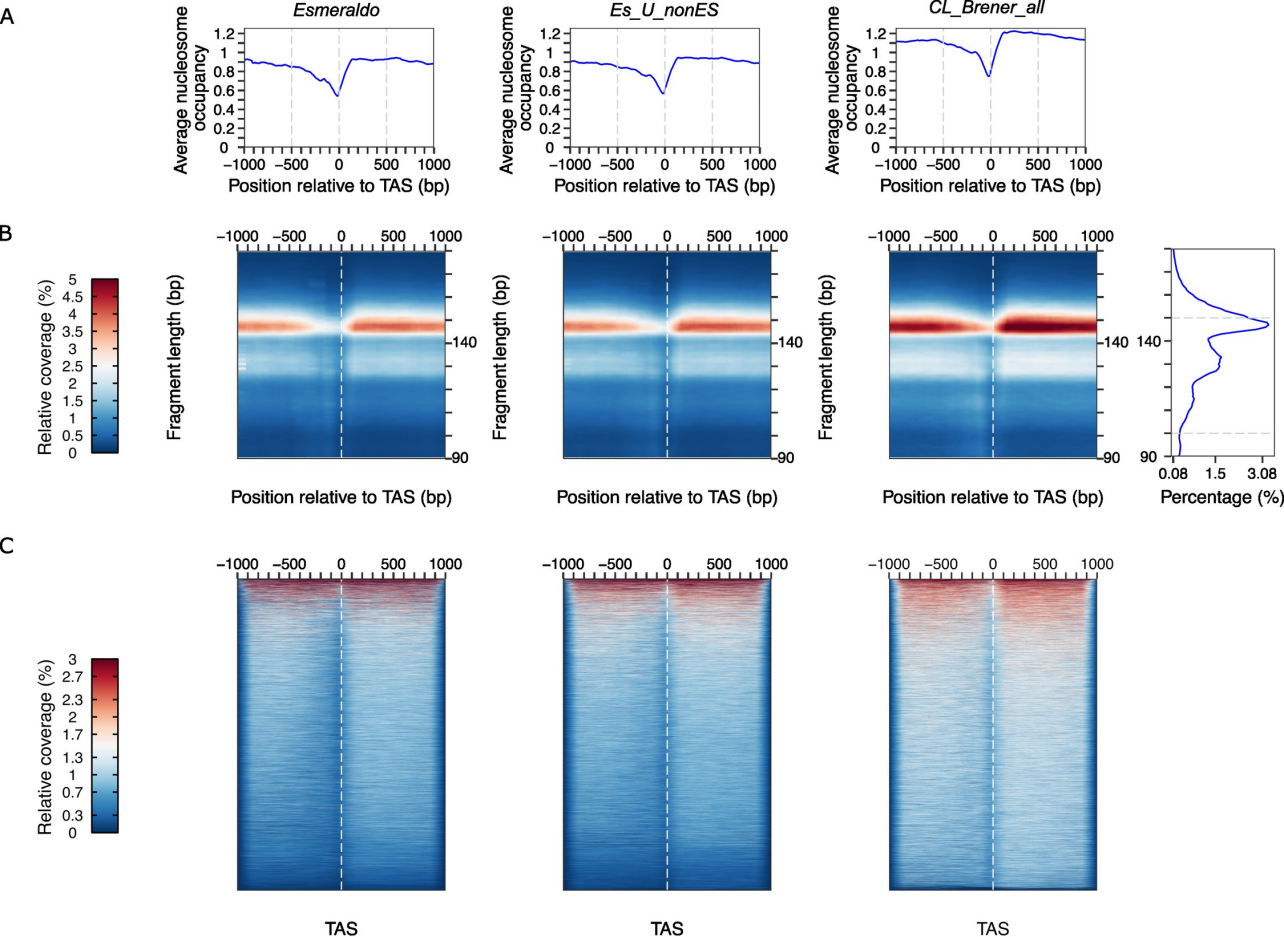
Using *CL Brener_all* genome prevents artefactual signals and improves nucleosome detection. **(A)** Average nucleosome occupancy, **(B)** 2D occupancy plots and **(C)** heatmaps showing nucleosome density represented relative to TAS performed from trimmed reads for one representative data set (replicate 1) process with Bowtie2. Red: High nucleosome density; blue: low nucleosome density.

Then, we generated average nucleosome occupancy, 2D occupancy plots and heatmaps for disaggregated regions around every TAS only for the S chromosomes but using the bam files generated from all the alternative alignments described above (Fig 4 and Fig S6 in S3 File). As previously described, we observed a mild depression upstream of the reference point in the average and the 2D Occupancy plots (Fig 4A and 4B). Moreover, the 2D plots allowed us to confirm that the size of the represented DNA is in the mononucleosome range. We also observe from 2D plots that we obtained noiseless representations when using *CL Brener_all* genome either with Bowtie2 or HISAT2, and *Es_U_nonEs* genome with Bowtie2 (S6 Fig in S3 File). However, when aligning the data to *Es_U_nonEs* with HISAT2 or to the *Esmeraldo* genome with either tool, some artefacts appeared, as denoted by the ladders of yellow bands of high or low molecular weight in the heatmaps.

Furthermore, we also represented the data into heatmaps for 1 kb window relative TAS but showing each region around every TAS in the y axis (Fig 4C). We could observe that only when using *Cl Brener_all* genome we can detect nucleosome signals around TAS higher than the background. Additionally, with this representation we could observe for the first time that nucleosome organization around TAS is not just an average but is conserved in most of the genome.

These observations highlight the importance of using the *CL Brener_all* genome as a reference for cleaner results even when the analysis will be focused only on one haplotype.

### Extending the use of the method to a non-hybrid strain

To explore the usefulness of the method described in S1 File beyond CL Brener strain, we performed the experiment with exponentially growing epimastigotes of the Sylvio-X10 strain. On one hand, we demonstrated that the experimental procedure is easily adjustable to a different *T*. *cruzi* strain as shown in the agarose gels for the replicated experiments (S7A Fig in S3 File). Upon sequencing the samples, we verified that the length of the sequenced DNA molecules is in the desired range, we obtained a significant number of reads for both replicates and we confirmed the reproducibility of the replicated experiments (S7B-S7D Fig in S3 File).

**Fig 5:**
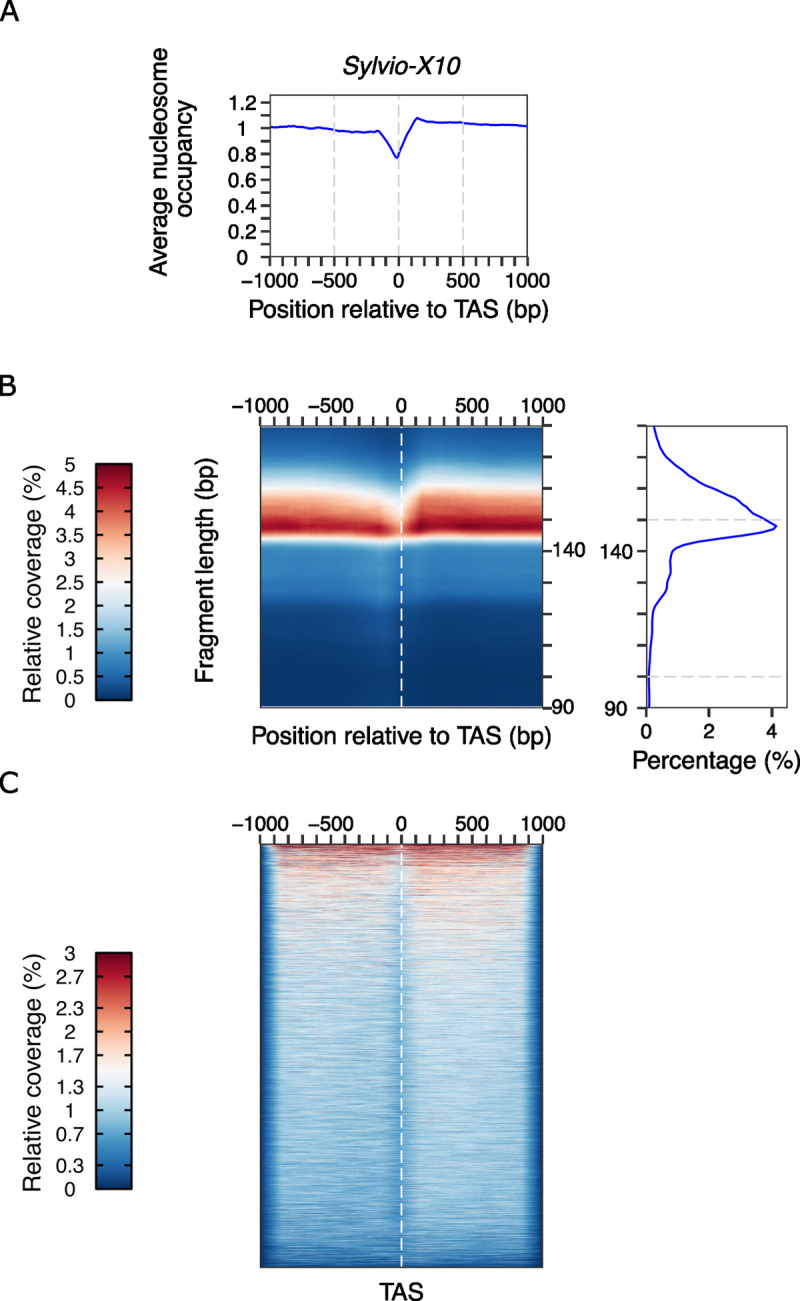
MNase-seq experimental and analytical approach can be applied to non-hybrid strains. **(A)** Average nucleosome occupancy, **(B)** 2D occupancy plots and **(C)** heatmaps showing nucleosome density represented relative to TAS. Red: High nucleosome density; blue: low nucleosome density. TAS predictions were made with UTRme using five transcriptomic data sets previously published (SRR2177699, SRR9202394, SRR2177820, SRR2180340, SRR2177823 [33].

On the other hand, we predicted the most likely TAS for Sylvio-X10 as described above and we represented average nucleosome occupancy, 2D occupancy plots and heatmaps for disaggregated regions around the TAS (Fig 5). Resembling CL Brener chromatin landscape, we observed a mild depletion of nucleosomes upstream of the reference point in the average and the 2D Occupancy plots (Fig 5A and 5B). Moreover, the 2D plots allowed us to confirm that the size of the represented DNA is in the mononucleosome range.

Furthermore, we also represented the data into heatmaps for 1 kb window relative TAS but showing each region around the predicted TAS in the y axis (Fig 5C) resembling the observations made for CL Brener strain. Therefore, the use of our method can be easily extended to any *T*. *cruzi* strain.

## Discussion

In this work, we described a high-resolution method for genome-wide mapping of nucleosomes in *T*. *cruzi* epimastigotes providing an upgraded step by step workflow for the experimental approach. Moreover, we performed a categorical informatic analysis (Fig 1B). We have provided the experimental details and an informatics pipeline which are easily adaptable to any *T*. *cruzi* strain.

A good MNase-seq protocol for *T*. *cruzi* was previously published [9]. Here, we upgraded it by providing cautious experimental details and careful considerations to perform the informatic analysis. In this regard, we first tested cell permeabilization as previously described for *T*. *brucei* using digitonin [14], but we encountered a huge variability in chromatin recovery among replicates. Hence, we switched to the protocol detailed here. Summarizing some technical advice, it is very important to avoid the use of vortex at any step of the procedure. Every solution must be prepared in advance, except when using PMSF, 2-mercaptoethanol or protease inhibitors that should be added fresh to the solutions. The range of MNase to be tested is only a suggestion. We recommend performing a titration in every replicate experiment, as described in S1 Fig in S3 File, since the level of digestion achieved is somewhat unpredictable and depends on many variables such as permeabilization efficiency, reaction conditions and the experience of the operator. In this regard, it is a good advice to aliquot the parasite sample right after cell permeabilization, instead of later in the protocol when the sample becomes more difficult to pipet. To make comparisons between different conditions, it is important to choose samples with similar levels of digestion.

In earlier work, samples were purified from a gel before library preparation and paired-end sequencing [13]. However, purification is not necessary given that size sorting can be done *in silico* afterwards. Moreover, it can be useful to count on the whole data including additional DNA lengths (e.g.: di-nucleosome bands) present in the sample for further analysis as previously described [19].

To properly assign the location of nucleosomes in the genome, it is extremely important to use paired-end technology for DNA sequencing. In this work, we obtained a remarkably good number of reads for each replicate experiment (S2C Fig in S3 File). Although it could be tempting, replicate data should never be combined for analysis because comparison of biological replicates is essential to assess reproducibility. Indeed, even though we corroborated the reproducibility between replicate experiments, by representing the scatter plot for read counts and obtaining a Pearson correlation coefficient of 0.97 for CL Brener starin and 0.98 for Sylvio-X10 strain (S2D and S7D Fig in S3 File respectively), we performed the whole analysis for each replicate separately.

Ideally, the strain of choice for high throughput studies should count on a well assembled and annotated genome. The first *T*. *cruzi* genome to be sequenced was the CL Brener strain [34]. Afterwards, the genomes of several other *T*. *cruzi* strains have been sequenced, assembled, and collected in the TriTryp database (https://tritrypdb.org/tritrypdb/). Unfortunately, most of them have been sequenced with short sequence read technologies, making accurate annotation almost impossible considering the high content of repetitive sequences of the *T*. *cruzi* genome. With the advent of long read technologies, such as Nanopore or PacBio, more reliable assemblies have been possible [35–37]. At the onset of this work, no genome sequence for any *T*. *cruzi* strain was of outstanding quality and CL Brener genome was one of the few sequences available. Hence, we chose CL Brener for counting on a sequenced genome and for being the reference strain and widely used by many researchers. Despite the CL Brener genome being poorly assembled and annotated, we developed a careful workflow considering these caveats for cautious conclusions.

Accordingly, the data should be aligned to the best genome version of the strain in which the experiment was performed. Here, we showed that using only the Esmeraldo-like haplotype or even combined with the non-Esmeraldo-like haplotype as reference genome, constitutes a blunder, introducing noise to the analytical process (Figs 3 and 4 and S4-S6 Figs in S3 File). In this regard, the genome regions encoding for multicopy gen families and the ends of the chromosomes have the greatest uncertainty in proper assembly, coincidental with the spots where spurious signals were observed when using *Esmeraldo* or *Esmeraldo_U_nonEs* genome as illustrated in Fig 3.

For in depth examination, it is a good decision to focus on the Esmeraldo-like haplotype since many studies and predictions from different labs have been based on this portion of the genome and it is useful for comparisons. However, the genomic data should be first aligned to the whole genome, *CL Brener_all*, as described in Fig 4 and S6 and S7 Figs in S3 File.

Given the complex characteristics of the hybrid genome, the performance of the widely used aligner Bowtie2 was compared with HISAT2. Deciding which are the best parameters to use, represents a conundrum due to the high content of repetitive sequences present in *T*. *cruzi* genome and the limited information about the exact percentage of these regions. From the statistics of alignments summarized in Table 1 and S1 Table in S3 File, we observed that the overall alignment was somewhat higher when using Bowtie2, while HISAT2 was discarding some repetitive reads being more restrictive for ambiguous alignments. Particularly, HISAT2 was more efficient at resolving replicated reads providing a bigger proportion of uniquely aligned reads with any reference genome tested. Unfortunately, due to the uncertainty about the exact content of repetitive regions present in the CL Brener genome, it is almost impossible to ensure which aligner is achieving a more precise result. However, from the average analysis and heatmaps presented in this work (Fig 4 and S6 Fig in S3 File), we concluded that both tools provide similar results when performing broad analysis. Additionally, careful examination on IGV shows that the alignment with HISAT2 results in a few regions with particularly high nucleosome density, even when choosing *CL Brener_all* genome. This unusually high nucleosome density appears only in a few regions, in general coincident with multi copy gene families such as MASP, and most likely correspond to repetitive regions that were collapsed during genome annotation. Therefore, using either of these aligners with the *CL Brener_all* genome is a good choice, but their limitations might be considered for careful conclusions in each case. For global analysis Bowtie2 is the tool of choice for providing the biggest overall alignment, but when the analysis needs to be focused on uniquely aligned reads or single copy genes families HISAT2 could be an overcoming alternative.

Finally, the critical point is given by the choice of the reference genome. Using only the Esmeraldo-like haplotype represents a simplification that could not only lead to artefactual signal detections (Figs 3 and 4B) but can also prevent the proper uncovering of nucleosome signals relative to the background (Fig 4C) misleading the chromatin organization analysis. Although the advent of an improved version of CL Brener genome is expectable in the community, in the meantime, the use of *CL_Brener_all_genome* can bring us closer to more reliable interpretations.

Moreover, we show that the average nucleosome organization around trans-splicing acceptor site described before for CL Brener [9], is not just an average since the same chromatin pattern is detected for most of the represented regions (Fig 4 and S5 Fig in S3 File). Furthermore, we observed this is also true for Sylvio-X10 strain (Fig 5 and S7 Fig in S3 File) suggesting that it is possibly a common characteristic for every *T*. *cruzi* strain.

## Conclusions

In the last few years, the relevance of chromatin studies in trypanosomes has become more prominent. Here, we provide an updated experimental protocol and a robust informatic analysis. Our updated method not only gives a step by step experimental and analytical workflow for MNase-seq but is relevant to any high throughput study performed with the CL Brener strain or any hybrid one that might require the alignment to a reference genome. Moreover, we demonstrate that it could be easily adaptable to non-hybrid strains as well.

It has been described that at least 50% of the CL Brener genome contains repetitive sequences and the two haplotypes differ by more than 5%. Additional work is still needed to improve the accuracy of genome assembly and annotations for CL Brener and other *T*. *cruzi* strains to get more certainty in the analysis of high-throughput studies.

## Supporting information

S1 File Step-by-step protocol for MNase digestion of *T*. *cruzi* chromatin and informatic analysis. (PDF)

S2 File List and genome coordinates of TAS predictions for Esmeraldo-like haplotype. (GFF)

S3 File It containing supporting figures and S2 Table. (PDF)
