# Supplementary material for: Improving genome-wide mapping of nucleosomes in Trypanosome cruzi
Source: PLoS One. 2023 Nov 21;18(11):e0293809. doi: 10.1371/journal.pone.0293809 (PMC10662739; doi:10.1371/journal.pone.0293809)
Supplement: S1 File — (PDF) [file pone.0293809.s001.pdf]

protocols.io

VERSION 2

OCT 24, 2023

OPEN ACCESS

**DOI:**
dx.doi.org/10.17504/protocols.io.6qpvr4w83gmk/v2

**External link:**
https://github.com/paulati/nucleosome; BioRxiv doi: 10.1101/2021.07.02.450927

# Improving genome-wide mapping of nucleosomes in Trypanosome cruzi. V.2

Paula Beati[1], Milena Massimino Stepñicka[1], Salomé C. Vilchez Larrea[1,2], Pablo Smircich[3,4], Guillermo Daniel Alonso[1,2], Josefina Ocampo[1]

[1]Instituto de Investigaciones en Ingeniería Genética y Biología Molecular, Consejo Nacional de Investigaciones Científicas y Técnicas, Vuelta de Obligado 2490, C1428ADN Buenos Aires, Argentina;
[2]Departamento de Fisiología, Biología Molecular y Celular, Facultad de Ciencias Exactas y Naturales, Universidad de Buenos Aires, Buenos Aires, Argentina;
[3]Laboratorio de Bioinformática, Departamento de Genómica, Instituto de Investigaciones Biológicas Clemente Estable (IIBCE), Montevideo, Uruguay;
[4]Sección Genómica Funcional, Facultad de Ciencias, Universidad de la República (UdelaR), Montevideo, Uruguay.

Milena Massimino Stepñicka: Former member;

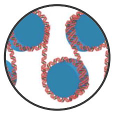

Josefina Ocampo

**Protocol Citation:** Paula Beati, Milena Massimino Stepñicka, Salomé C. Vilchez Larrea, Pablo Smircich, Guillermo Daniel Alonso, Josefina Ocampo 2023. Improving genome-wide mapping of nucleosomes in Trypanosome cruzi.. **protocols.io** https://dx.doi.org/10.17504/protocols.io.6qpvr4w83gmk/v2Version created by Josefina Ocampo

**License:** This is an open access protocol distributed under the terms of the Creative Commons Attribution License, which permits unrestricted use, distribution, and reproduction in any medium, provided the original author and source are credited

**Protocol status:** Working
We use this protocol and it's working

**Created:** Oct 18, 2023

**Last Modified:** Oct 24, 2023

**PROTOCOL integer ID:** 89570

**Keywords:** Chromatin; Nucleosomes; Micrococcal nuclease; genome-wide mapping; Trypanosoma cruzi

**Funders Acknowledgement:**
Agencia Nacional de Promoción Científica y Tecnológica
Grant ID: PICT 2015-0898
Agencia Nacional de Promoción Científica y Tecnológica
Grant ID: PICT 2018-00621
Agencia Nacional de Promoción Científica y Tecnológica
Grant ID: PICT 2020-00473

## ABSTRACT

In *Trypanosoma cruzi* DNA is packaged into chromatin by octamers of histone proteins that form nucleosomes. Transcription of protein coding genes in trypanosomes is constitutive producing polycistronic units and gene expression is primarily regulated post-transcriptionally. However, chromatin organization influences DNA dependent processes. Hence, determining nucleosome position is of uppermost importance to understand the peculiarities found in trypanosomes. To map nucleosomes genome-wide in several organisms, digestion of chromatin with micrococcal nuclease followed by deep sequencing has been applied. Nonetheless, the special requirements for cell manipulation and the uniqueness of the chromatin organization in trypanosomes entails a customized analytical approach. In this work, we adjusted this broadly used method to the hybrid reference strain, CL Brener. Particularly, we implemented an exhaustive and thorough computational workflow to overcome the difficulties imposed by this complex genome. We tested the performance of two aligners, Bowtie2 and HISAT2, and discuss their advantages and caveats. Specifically, we highlight the relevance of using the whole genome as a reference instead of the commonly used Esmeraldo-like haplotype to avoid spurious alignments. Moreover, we show that using the whole genome refines the average nucleosome representation, but also the quality of mapping for every region represented. Furthermore, we provide a source code for the construction of 2D plots and heatmaps which are easy to adapt to any *T. cruzi* strain.

## GUIDELINES

Do not vortex at any step of the protocol.
Add protease inhibitors fresh.
The range of MNase to be tested is only a suggestion. We recommend performing a titration in every replicate experiment.

## MATERIALS

**Special material for MNase digestion**
Micrococcal Nuclease (MNase), Worthington Biochemical Corporation, Cat No: R1A12424
**Special material for library preparation**
PrePCR kit (New England Biolabs, Ipswich, MA, US)
Qiagen PCR column (Qiagen, Hilden, Germany)
NEBNext Ultra DNA Library Prep Kit for Illumina and NEBNext Multiplex Oligos for Illumina (New England Biolabs, Ipswich, MA, US).
Agencourt AMPure XP beads (Beckman-Coulter, Brea, CA, US)

## BEFORE START INSTRUCTIONS

Prepare every solution in advance before you start with the MNase digestion.
**Incomplete lysis solution**
(1 mM L-glutamine, 250 mM sucrose, 2.5 mM $CaCl_2$, 1 mM phenylmethylsulfonyl fluoride (PMSF)).
**Complete lysis Solution**
(10 ml incomplete Lysis Solution, 0.01% Triton X 100, 1 mM PMSF).
**MNase digestion buffer**
(10 mM HEPES, 35 mM NaCl, 500 µM $MgCl_2$, 500 µM $CaCl_2$, 5 mM 2-mercaptoethanol and protease inhibitors)
**MNase stock solution**
Disolve Micrococcal Nuclease (MNase), Worthington Biochemical Corporation, Cat No: R1A12424 25,271 U/mg P, 82% protein, 45 Ku in 4.5 ml MNase storage buffer, aliquot out in 9x 500ml. Stored at -80°C.
**MNase storage buffer**
(5 mM Na-phosphate, pH7.0, 0.025mM $CaCl_{2)}$
**MNase Stop Solution**
(180 mM EDTA, 7.2 % SDS prepared in $H_2O$)

## Epimastigote cultivation and harvest

1 CL Brener epimastigotes were grown at 28 °C in liver infusion tryptose (LIT) medium (5 g.l-1 liver infusion, 5 g.l-1 bacto-tryptose, 68 mM NaCl, 5.3 mM KCl, 22 mM $Na_2HPO_4$, 0.2% (w/v) glucose, and 0.002 % (w/v) hemin) supplemented with 10 % (v/v) fetal calf serum (FCS), 100 U/ml penicillin and 100 mg. $l^{-1}$streptomycin. Cell density was maintained between $1\times10^6$and $1\times10^8$ cells.$ml^{-1}$by sub-culturing parasites every 6-7 días.

## MNase digestion of T. cruzi epimastigote chromatin

2 Pellet $10e^8$ late exponentially growing epimastigotes per reaction (normally 7) by centrifugation at 1700 xg for 5 minutes at Room temperature. Wash the cells with 10 ml Incomplete Lysis Solution (1 mM L-glutamine, 250 mM sucrose, 2.5 mM $CaCl_2$, 1 mM phenylmethylsulfonyl fluoride (PMSF)).

3 Centrifuge at 1700 xg for 5 minutes and discard supernatant.

4 Permeabilize cells with 800 µl Complete Lysis Solution (10 ml Incomplete Lysis Solution, 0.01% Triton X 100, 1 mM PMSF).

5 Divide the material into 7 aliquots of 100 µl each into 1.5 ml microcentrifuge tubes.

6 Centrifuge at 1700 xg for 5 minutes and discard supernatant.

7 Wash the pellets with 100 µl Incomplete lysis solution.

8 Centrifuge at 1700 xg por 5 minutos.

9 Resuspend the pellets in MNase Digestion Buffer (10 mM HEPES, 35 mM NaCl, 500 µM $MgCl_2$, 500 µM $CaCl_2$, 5 mM 2-mercaptoethanol and protease inhibitors). Pre-warm the buffer in a 25 °C water bath for 2 min before resuspension

10 Add increasing amounts of MNase (10 U/µl) to each tube, for example: 0, 1, 3.5, 7, 10, 20, 40 µl, and mix thoroughly without vortexing. Incubate the

digests at 25 °C for 5 min. Stop the reaction by adding 50 µl MNase Stop Solution (180 mM EDTA, 7.2 % SDS prepared in $H_2O$) to each digestion, mixing, and leaving at room temperature for 5 min.

11 Add 50 µl de 10 % SDS and mix (final SDS ˜1.8% in ˜500 µl final volume).

12 Add 250 µl of 3M KOAc (˜1 M final in ~750 µl final volume) and mix.

13 Extract the DNA by adding 750 µl chloroform, mixing and spinning in a microcentrifuge (5min at top speed). Repeat extraction.

14 Transfer the supernatants to new 1.5-ml tubes and add 0.7 vol. isopropanol to precipitate the DNA. Mix and leave at -20 °C Overnight

15 Pellet the DNA by spinning in a microcentrifuge (30 min at top speed).

16 Discard the supernatant. Wash the pellet once with 0.6 ml 70 % EtOH and spin in a microcentrifuge (5 min at top speed).

17 Discard the supernatant and dissolve each pellet in 50 µl of 10 mM Tris HCl pH 8, 2 mM EDTA with 100 µg/ml of RNAsa. Incubate the samples at 37 °C for 2 hours.

## Paired-end sequencing

18 Paired-end libraries were prepared as described before (1,2).
Briefly, once the DNA samples with the appropriate level of digestion were selected, any nicks were repaired using the PrePCR kit (New England Biolabs, Ipswich, MA, US), purified using a Qiagen PCR column (Qiagen, Hilden, Germany) and eluted with 50 µl TE buffer (10 mM Tris-Cl pH8, 0.1 mM EDTA). Modification of the 5' and 3' ends of the nucleosomal DNA and adapter ligation were performed with the NEBNext Ultra DNA Library Prep Kit for Illumina and NEBNext Multiplex Oligos for Illumina (New England Biolabs, Ipswich, MA, US). Agencourt AMPure XP beads (Beckman-Coulter, Brea, CA, US) were used to purify the DNA after adaptor ligation and PCR products before sequencing as described (1,2).

The beads were used in a 1:1 volume ratio, which removes adaptors and any primer dimers but retains nucleosomal DNA ligated to adaptors and anything larger. For sequencing reactions, either Illumina Next-seq 500 or Hi-seq 2000 were used to obtain pairs of 50 nucleotide (nt) reads.

## Data files and statistical analysis

19 Paired-end reads are normally provided in the fastq file format containing the raw reads. To do quality control checks on raw data, fastQC analysis was performed. The fastQC reports of the current data could be found at https://github.com/paulati/nucleosome/tree/master/output/qc_output. During this step over-represented sequences (ORS) were detected and trimmed out to avoid the generation of artefacts during the analysis. ORS in general are 50 nts long,when working with 50 nts reads,and correspond to adaptors or primers used for library constructionas detailed in the fastQC report. In the case of *T. cruzi,*due to the repetitive nature of its genome, some ORS occasionally contain runs of repetitive sequences. ORS were removed when present producing trimmed fastq files using the Cutadapt tool (3), containing trimmed raw reads that from now on we will refer to as "*trimmed reads*". Given that this step is regularly omitted when analyzing MNase-seq data from model organisms, to evaluate if it constitutes an improvement in the analysis of *T. cruzi*, we pursued the following steps in parallel using raw reads and trimmed reads.

## Sequence alignment and reference genome

20 Bowtie2 (4)and HISAT2 (5) were used to align either the raw or the trimmed 50 nt paired-end reads, to the corresponding reference genome. To compare the performance of these tools, the same parameters values were set for both tools. In particular, both tools have a parameter called score-min, defined as a function of the read length, that is the minimum score needed for an alignment to be considered “valid”.
For Bowtie2, the end-to-end mode was used with default settings, commonly used for model organisms, where the score-min is specified by `L, -0.6, -0.6`. This, establish a linear function f (x) = -0.6 + -0.6 * x, where x is the read length. In this case, x is 50 nts, which implies it accepts scores bigger than -30.6. In the case of HISAT2, the default settings are more restrictive than those for Bowtie2 with `L, 0, -0,2`. To make a fair comparison between both tools, the exact same score-min used for Bowtie2 was set for HISAT2.

Esmeraldo-like is considered the reference genome. In this work, this was used as a reference and two additional reference genomes were generated for comparison. One, combining Esmeraldo-like and non-Esmeraldo-like haplotypes, designated *Es_U_nonEs*. The second one, combining Esmeraldo-like, non-Esmeraldo-like and "extra" regions that are still not assigned to any haplotype and are annotated separately. This wider genome, is designated *Cl Brener_all*. Versions TriTryp46 of the respective genomes were used as a reference for informatic analysis in every case.

## Binary file generation

21 Bigwig files containing information of nucleosome occupancy and nucleosome position were generated. Occupancy (coverage) maps were built by counting the number of times that a base pair was occupied by a nucleosome, while position maps counted the number of times each particular nucleosome sequence was present in the data set. The midpoint of the nucleosome, called the dyad, was represented as previously described (6–8). To observe more clearly the nucleosomes in the occupancy maps and to be more accurate in the estimation of the position maps, the analysis was restricted to those fragments that belong to the nucleosome size range (120-180 bp) and is normalized by summing all the nucleosome sequences covering a nucleotide and dividing that number by the average number of nucleosome sequences per base pair across the genome.

## Prediction of the trans-splicing acceptor site

22 The 5´untranslated regions (5'UTR) were predicted with UTRme (9). Given the lack of transcriptomic data for CL Brener epimastigotes, the predictions were based on available RNA-seq data for the Y strain (10) using TriTryp46 Esmeraldo-like genome as a reference. As an approximation of the trans-splicing acceptor site (TAS), the 5' end of the 5'UTR region predicted with the best score were used.

## Average plots and 2D occupancy plots

23 Average representation of the data and 2D Occupancy plots were performed as previously described (11,12), with some modifications. Average nucleosome determinations were performed from the alignments

generated in all the alternative alignments and represented relative to the predicted TAS in a 1kb window. Briefly, even when the total number of reads was previously aligned to the *Es_U_nonEs*or the *CL Brener_all* genomes, for this analysis only the average nucleosome occupancy coming from the reads that aligned to the Esmeraldo-like haplotype was represented relative to the predicted TAS.We define nucleosome density as the normalized percentage of occupancy. For 2D Occupancy plots, the nucleosome density was represented relative to the TAS in the x axis, while the size of the analyzed DNA fragments was represented in the y axis. For heatmaps showing every region around TAS, nucleosome density was represented in the same way in the x axis; while in the y axis, for each gene region, we computed the normalized total occupancy for the region as the normalized sum of occupancies for each position in the region, and then ordered the regions according to these descending values.

protocols.io
