# Supplementary material for: Improving genome-wide mapping of nucleosomes in Trypanosome cruzi
Source: PLoS One. 2023 Nov 21;18(11):e0293809. doi: 10.1371/journal.pone.0293809 (PMC10662739; doi:10.1371/journal.pone.0293809)
Supplement: S3 File — (PDF) [file pone.0293809.s003.pdf]

Genome-wide mapping of nucleosomes in *Trypanosoma cruzi* is improved by using a more appropriate reference genome.

Paula Beati¶, Milena Massimino Stepñicka ¶, Salomé Vilchez Larrea, Pablo Smircih, Guillermo D. Alonso* and Josefina Ocampo*

¶ These authors have contributed equally to this work and share first authorship.
* Correspondence: galonso@dna.uba.ar (G.D.A.); jocampo@dna.uba.ar (J.O.)

Supplementary Figures and Table

S1 Fig

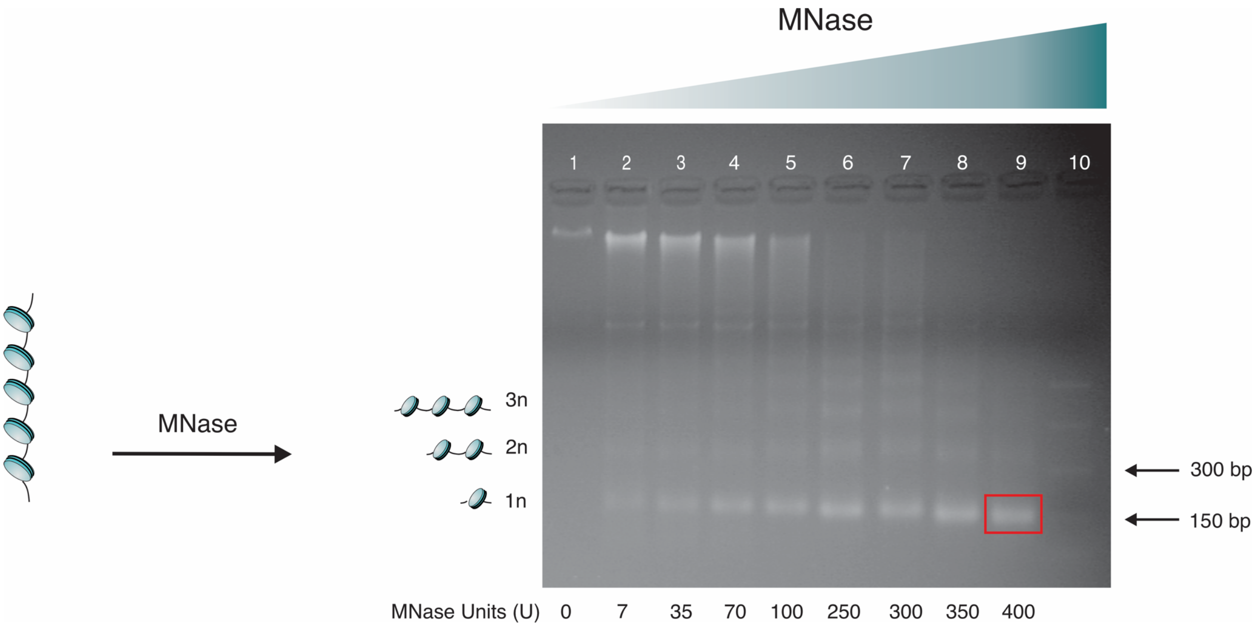

S1 Fig. Titration of *T. cruzi* chromatin with MNase. Chromatin digested with increasing amounts of MNase analyzed in a 2 % agarose gel (lanes 1-9). An example of the level of digestion of choice is highlighted in lane 9. PCR DNA marker (New England Biolabs, Ipswich, MA, US) was loaded in lane 10.

S2 Fig

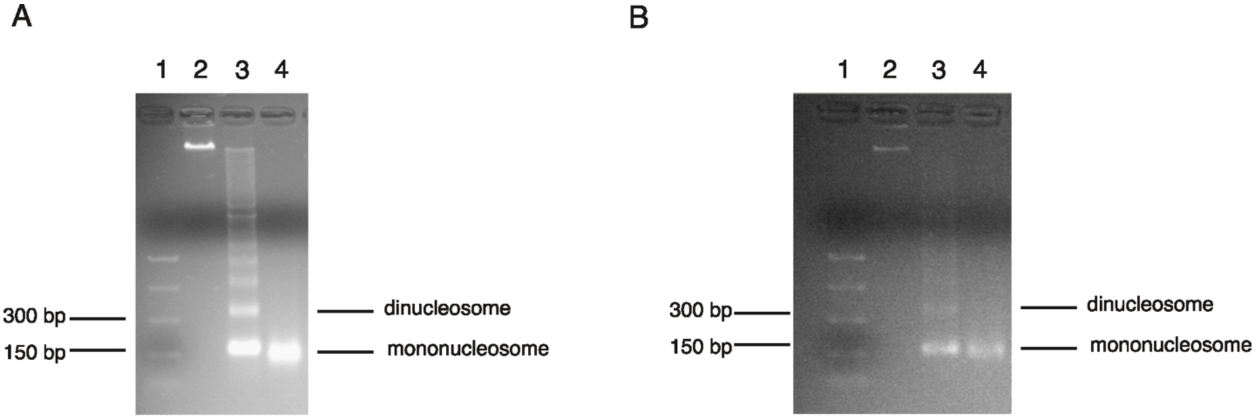

C

Samples	Number of reads
CL Brener replica 1	73875257
CL Brener replica 2	83417349

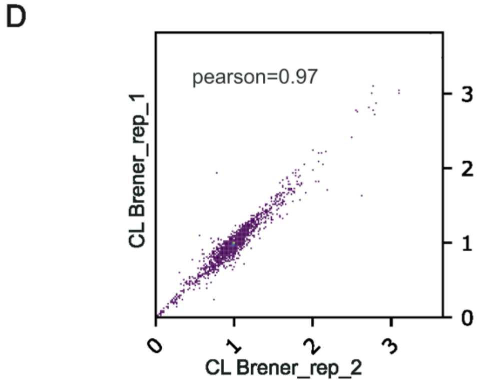

S2 Fig. Mononucleosome sample, total number of paired-reads, and reproducibility. Chromatin digestions from epimastigotes of the CL Brener strain for **(A)** replicate 1 and **(B)** replicate 2 experiments analyzed in a 2 % agarose gel. The samples loaded in lane 4 respectively were used for the experiments. PCR DNA (New England Biolabs, Ipswich, MA, US) marker was loaded in lane 1 in both gels. **(C)** Total number of paired-reads obtained in each replicate experiment. (D) Scatter plot of read counts for each dataset (biological replicates) from CL Brener epimastigotes showing the Spearman correlation coefficient generated with DeepTools. CL Brener_rep1: replicate 1; CL Brener_rep2: replicate 2.

S3 Fig

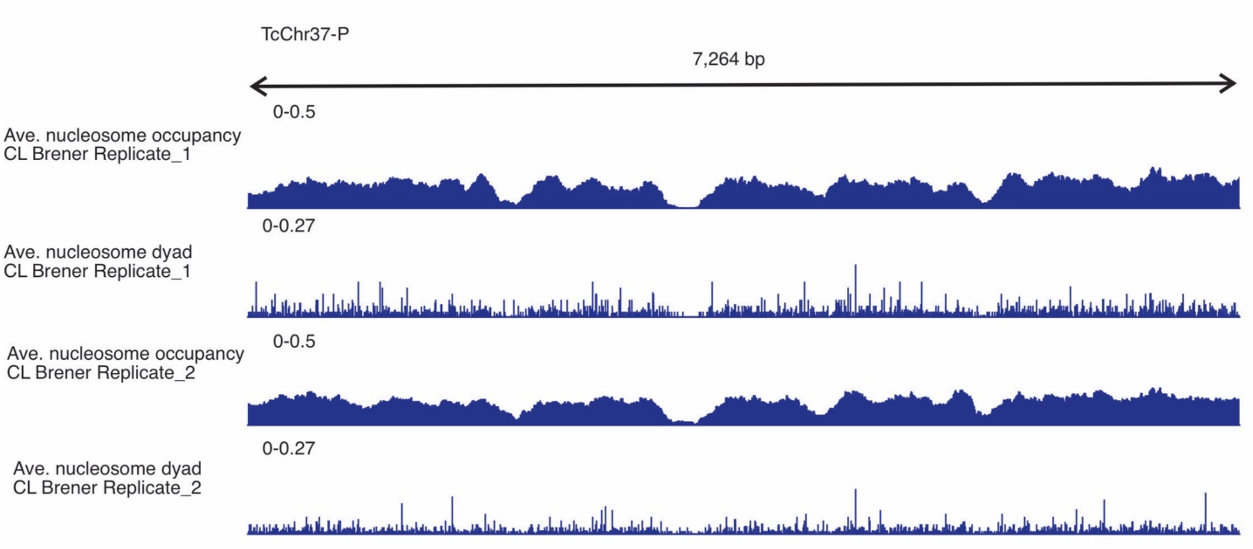

S3 Fig. Nucleosome occupancy and nucleosome position maps. Normalized nucleosome occupancy and nucleosome position maps are shown for a representative region of chromosome 37-P from de non Esmeraldo-like haplotype for both replicate experiments

S4 Fig (associated to Fig 3)

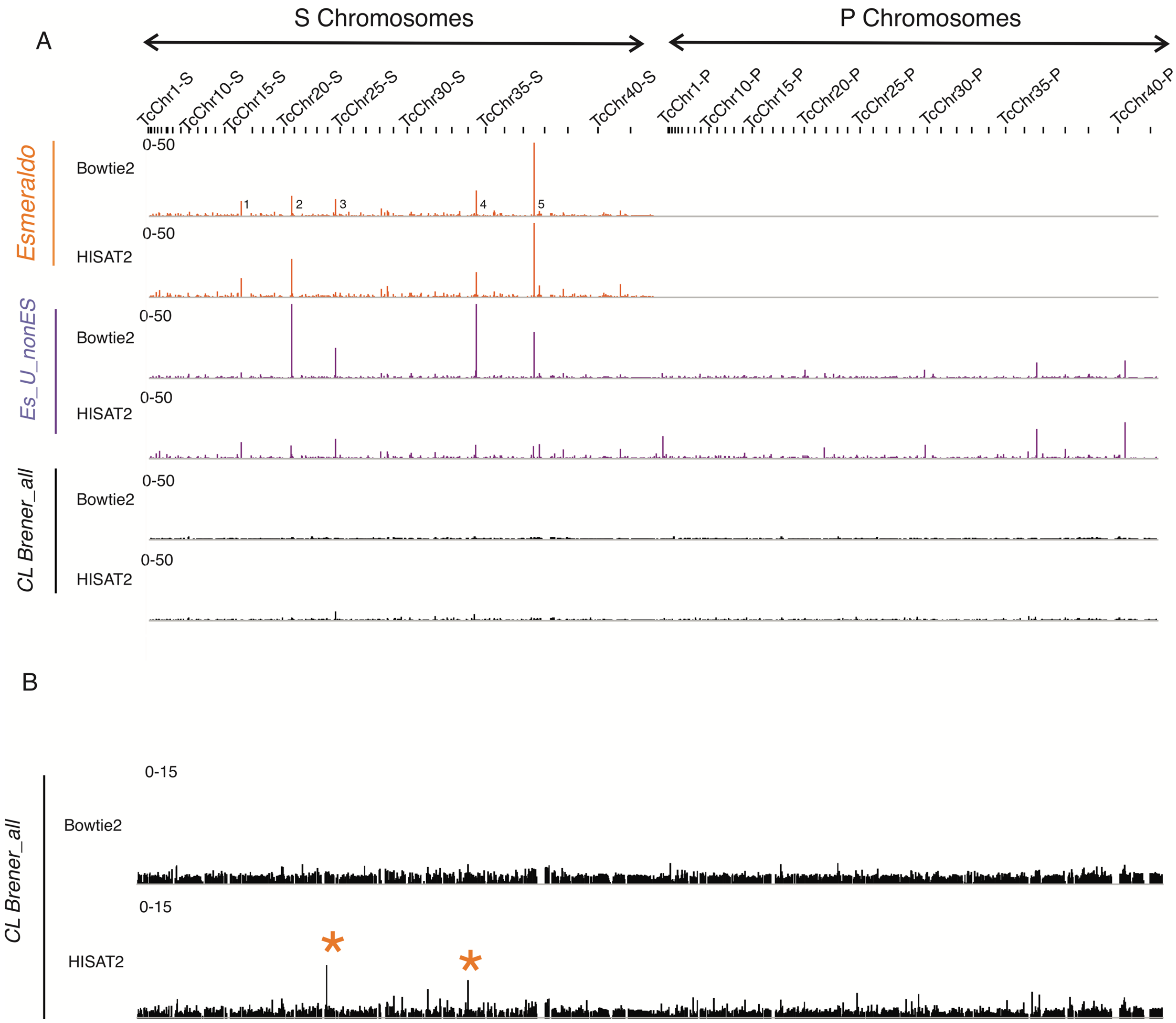

S4 Fig. Using *CL Brener_all* genome prevents spurious alignments. (A) IGV image for normalized nucleosome occupancy maps for the whole genome generated with raw reads from one representative data set (replicate 1) aligned to the *Esmeraldo* (orange), *Es_U_nonEs* (purple) and *CL Brener_all* (black) genomes respectively either with Bowtie2 or HISAT2. Numbers 1-5 in indicate the positions of the unusually high regions, being their genomic locations as follow: 1: TcChr15-S:394,542-407,393; 2: TcChr20-S:533,028-533,218; TcChr20-S:609,812-611,415 and TcChr20-S:655,789-656,728; 3: TcChr24-S:777,166-779,922, 4: TcChr34-S:1,062,764-1,065,764 and 5: TcChr37-S:1,199,919 and TcChr37-S:1,354,130-1,355,692". **(B)** Magnified IGV image for the nucleosome occupancy maps generated from data aligned to the *CL Brener_all* genome represented in **(C).** Orange (*) indicates the main artefacts introduced when using HISAT2.

S5 Fig (associated to Fig 3)

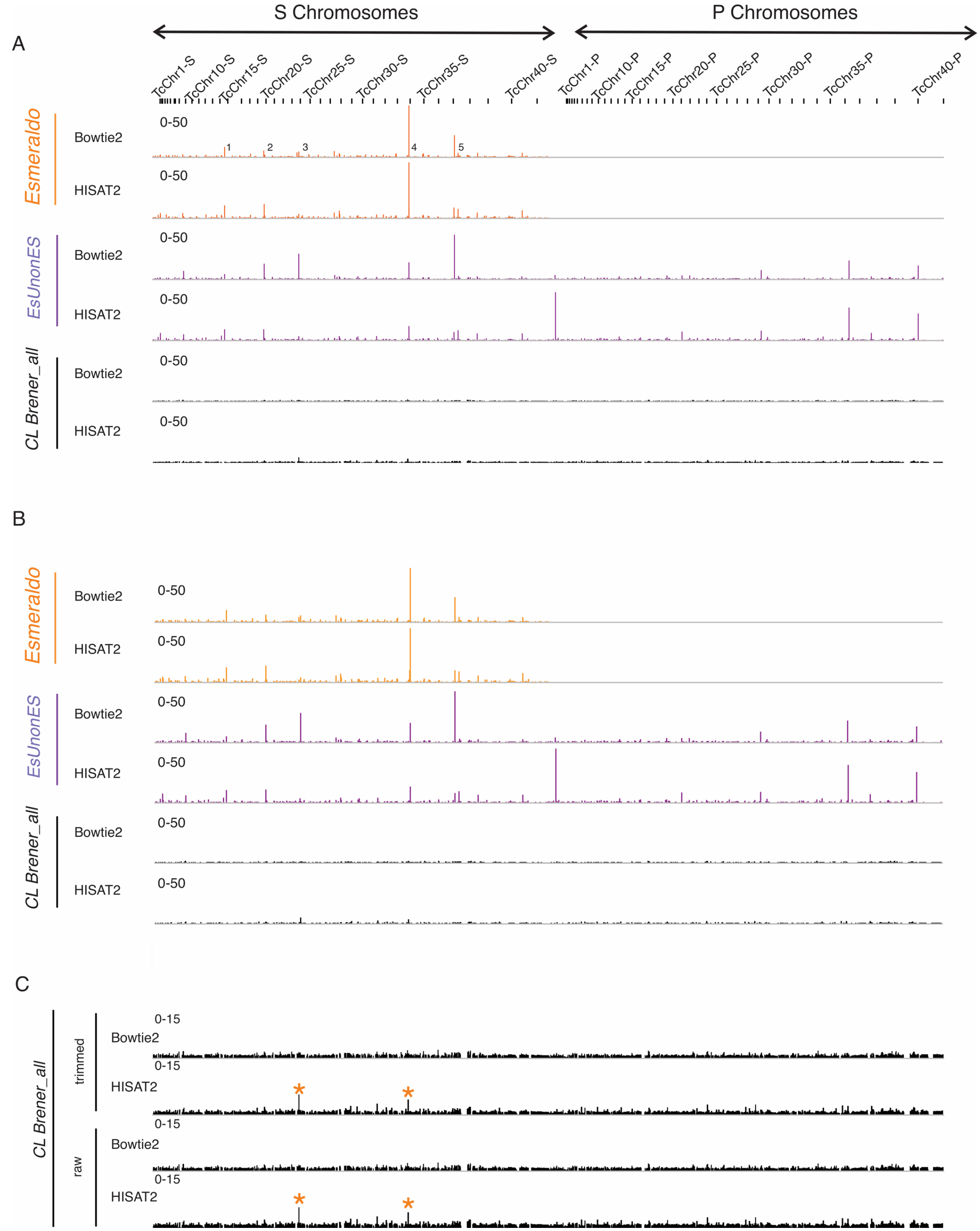

S5 Fig. The use of CL Brener_all genome prevents spurious alignments. IGV image of the whole genome for normalized nucleosome occupancy maps generated from **(A)** trimmed **(B)** and raw reads for one representative data set (replicate 2) aligned to the *Esmeraldo* (orange), *Es_U_nonEs* (purple) and *CL Brener_all* (black) genomes respectively either with Bowtie2 or HISAT2. Numbers 1-5 in (a) indicate the positions of the unusually high regions, being their genomic locations as follow: 1: TcChr15-S:394,621-407,444; 2: TcChr20-S:532,974-533,253; 609,848-611,485 and TcChr20-S:655,808-656,663; 3: TcChr24-S:776,972-777,763, 4: TcChr34-S:1,062,859-1,065,565 and 5: TcChr37-S:1,200,137-1,207,104 and TcChr37-S:1,354,096-1,355,707. **(C)** Magnified IGV image for the nucleosome occupancy maps generated from data aligned to the *CL Brener_all* genome represented in (A) and (B). Orange (*) indicates the main artefacts introduced when using HISAT2.

S6 Fig (associated to Fig 4)

A

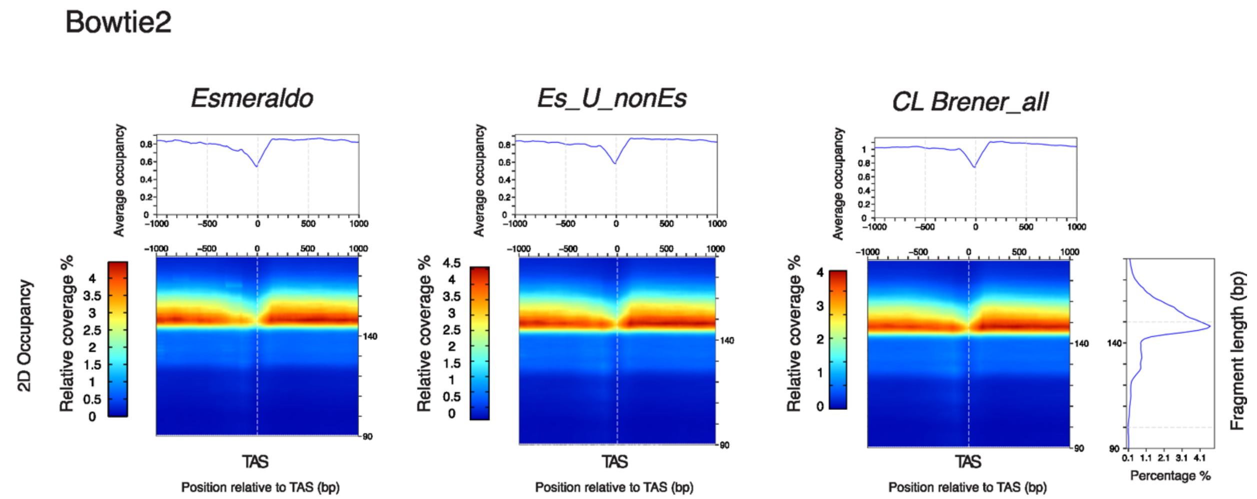

B

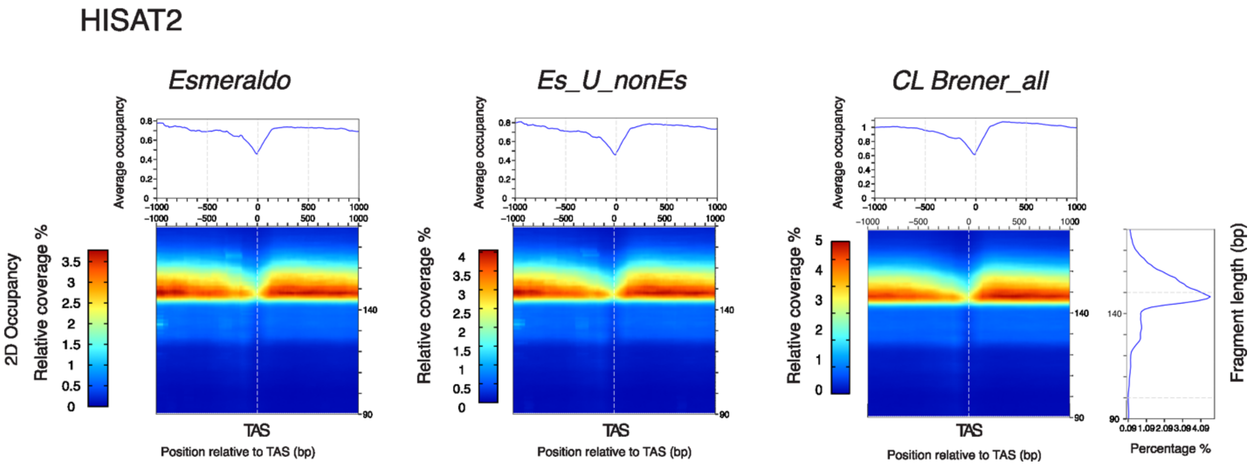

S6 Fig. Using *CL Brener_all* genome prevents artefactual signals. Average nucleosome occupancy and 2D occupancy plots performed from trimmed reads for one representative data set (replicate 2) process with either **(A)** Bowtie2 or **(B)** HISAT2. Red: High nucleosome density; blue: low nucleosome density.

S7 Fig (associated to Fig 5)

A

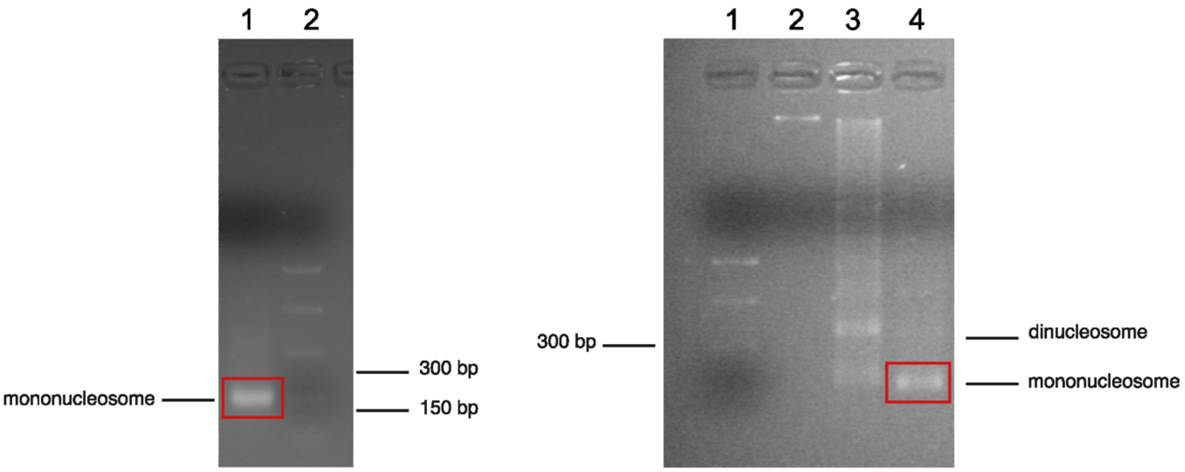

B

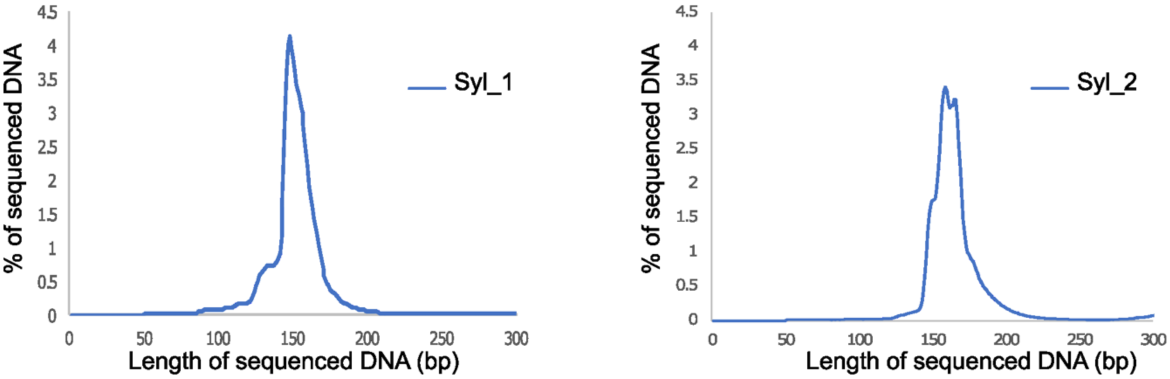

C

Samples	Number of reads
Sylvio-X10 replica 1	72874447
Sylvio-X10 replica 2	63293611

D

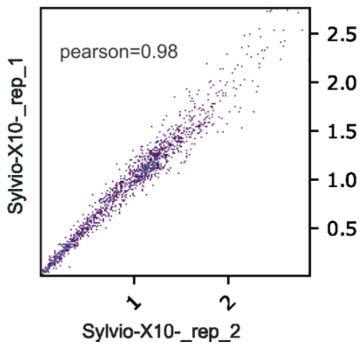

S7 Fig. Mononucleosome sample, total number of paired-reads and reproducibility for Sylvio-X10. (A) Chromatin digestions from epimastigotes of the Sylvio-X10 strain for replicate 1 (left panel) and replicate 2 (right panel) experiments analyzed in a 2% agarose gel. The samples squared in red were used for the experiments. PCR DNA (New England Biolabs, Ipswich, MA, US) marker was loaded in lane 1 in both gels. (C) Total number of paired-reads obtained in each replicate experiment after aligning raw reads to the reference genome Sylvio-X10 version 64 from TriTrypDB. (D) Scatter plot of read counts for each dataset (biological replicates) from CL Brener epimastigotes showing the Spearman correlation coefficient generated with DeepTools. CL Brener_rep1: replicate 1; CL Brener_rep2: replicate 2.

S1 table

CL Brener	Bowtie2	trimmed reads	genome	0 times	1 time	> time	overall
			Esmeraldo	43	24.7	32.31	57
			Es_U_nonEs	31.34	11.62	57.03	68.66
			CL Brener_all	23.3	10.3	66.4	76.7
		raw reads	genome	0 times	time	> time	overall
			Esmeraldo	43.98	24.25	31.76	56.02
			Es_U_nonEs	32.58	11.41	56.01	67.42
			CL Brener_all	24.68	10.11	65.21	75.32
	HISAT2	trimmed reads	genome	0 times	1 time	> time	overall
			Esmeraldo	43.07	45.09	11.84	56.93
			Es_U_nonEs	33.36	49.53	17.11	66.64
			CL Brener_all	30.96	44.75	24.28	69.04
		raw reads	genome	0 times	1 time	> time	overall
			Esmeraldo	44.09	44.29	11.62	55.91
			Es_U_nonEs	34.55	48.65	16.8	65.45
			CL Brener_all	32.2	43.95	23.84	67.8

S1 Table. Statistics of alignments. The percentage of alignments for the different paths tested with replicate 2 is summarized. Values obtained when using trimmed reads or raw aligned to the alternative genomes are shown.
